# Proposals and Comparisons from One-Sensor EEG and EOG Human-Machine Interfaces

**DOI:** 10.3390/s21062220

**Published:** 2021-03-22

**Authors:** Francisco Laport, Daniel Iglesia, Adriana Dapena, Paula M. Castro, Francisco J. Vazquez-Araujo

**Affiliations:** CITIC Research Center, University of A Coruña, Campus de Elviña, 15071 A Coruña, Spain; daniel.iglesia@udc.es (D.I.); adriana.dapena@udc.es (A.D.); paula.castro@udc.es (P.M.C.); fjvazquez@udc.es (F.J.V.-A.)

**Keywords:** human-machine interfaces, electroencephalography, electrooculography, P300, graphical user interface

## Abstract

Human-Machine Interfaces (HMI) allow users to interact with different devices such as computers or home elements. A key part in HMI is the design of simple non-invasive interfaces to capture the signals associated with the user’s intentions. In this work, we have designed two different approaches based on Electroencephalography (EEG) and Electrooculography (EOG). For both cases, signal acquisition is performed using only one electrode, which makes placement more comfortable compared to multi-channel systems. We have also developed a Graphical User Interface (GUI) that presents objects to the user using two paradigms—one-by-one objects or rows-columns of objects. Both interfaces and paradigms have been compared for several users considering interactions with home elements.

## 1. Introduction

During the last years, Human-Machine Interfaces (HMIs) and, especially, Brain-Computer Interfaces (BCIs), have become very active research fields with significant developments and advances in non-clinical areas such as entertainment, home automation or cognitive training [1]. BCI systems can be defined as the combination of hardware and software in a communication system that monitors the user’s cerebral activity and translates certain characteristics, corresponding to the user’s intentions, to commands for device control [2]. BCIs present a new communication channel with outer devices without the involvement of peripheral nerves and muscles, allowing users to interact with the environment without any physical activity and using only their thoughts. Therefore, it results in an extremely useful technology for patients with severe motor disabilities. Electroencephalography (EEG) is the most widely used technique for neuroimaging and brain signal acquisition for BCIs. This preference is mainly based on the non-invasive character of this technology, which implies a low risk for the users. Moreover, it shows several advantages such as its high portability and temporal resolution, its relatively low cost, and its ease of use [1,3,4]. The brain activity collected by the EEG encodes the users’ intentions, which can be translated into control commands for interacting with their surroundings. For this purpose, several brain signal patterns have been assessed, where Motor-Imagery (MI) synchronisation/desynchronisation, Steady-State Visual Evoked Potential (SSVEP) and P300 Evoked Potential (P300 EP) are the most common approaches [1].

The P300 EP is a positive deflection located in the parietal area of the cortex that occurs in the EEG brain activity 300ms after an infrequent or surprising auditory, visual or somatosensory stimuli [5,6,7]. The potential is usually evoked by the “oddball” paradigm, where several stimuli are presented to the subject and one of them is less frequent than the others. Based on this brain response, a specific action can be associated to that stimulus so it will be executed when the P300 is detected. The most common way to employ the P300 EP in BCIs is through visual stimulation, where different elements which users can interact with are, usually, randomly presented on a screen. Users must focus their attention on one of these infrequent elements so that when it appears, the P300 potential will be elicited and BCIs can detect and execute associated actions.

Voluntary eye movements such as blinking, saccades or fixation have also been used as control signals in different HMIs. The electrical signal produced by the eye activity can be measured using the Electrooculography (EOG) technique, also a non-invasive, portable, easy to use and affordable technology [8]. EOG signals are stronger in amplitude than the EEG ones, so they are easier to detect and more stable across users [9], which can be an advantage to improve the accuracy of the interface. Consequently, several HMIs have been proposed employing blinking movements as control signals [9,10]. The eye can be modelled as a dipole with its positive pole at the cornea and its negative pole at the retina [8]. When closing and opening the eyelids, that is, blinking, a vertical movement is produced in the eye, which causes a change in the dipole orientation and thus a change in the electric potential field measured by EOG. As a consequence, a blink presents a specific pattern captured by the EOG signal, characterized by two consecutive large peaks, positive and negative, respectively, corresponding to the closing and opening of the eye. These two large peaks are easily recognized from the recorded signal, so HMIs can detect them in order to execute associated actions.

Non-contact methods, such as Videooculography (VOG), have also been successfully applied for eye gaze analysis and blink detection to develop new HMIs [11,12]. These detection systems usually employ several cameras that record the user’s eyes and, applying image processing techniques and artificial vision algorithms, they can provide an accurate analysis of the eye state of the user [13,14,15]. However, in comparison with EOG techniques, these VOG methods have a high computational complexity derived from image analysis and classification. This makes it difficult to implement in Single-Board Computers (SBC) integrated into, for example, a smart home environment.

The use of EEG and EOG signals for interaction with home elements is a current challenge. Previous works have developed multi-sensor systems to capture brain or eye activity to detect the user’s intentions [16,17,18,19,20,21]. However, multi-sensor recording devices are usually large and cumbersome, which becomes a problem when used for several hours a day. Single-channel solutions have been proposed in order to overcome this issue [10,22,23,24]. Recently, we have shown that low-cost systems with only one input electrode are very useful for developing Internet of Things (IoT) applications in smart-home environments [25]. Keeping up with this idea, in this paper we develop and compare two systems focused on the interaction between users and home elements. The first system is based on the P300 evoked potential, whereas the second one uses eye blinks as control signals. We have developed a Graphical User Interface (GUI) where home elements are displayed in a matrix-form and presented using two different paradigms: (i) home elements are presented one-by-one; (ii) the elements of the same row/column are presented together. Our objective is to determine which combination of system and paradigm offers the best performance in terms of accuracy and time delay.

The paper is organized as follows. Section 2 summarizes some of the most important works related to the utilization of the P300 potential and voluntary blinks as control signals. Section 3 shows the two developed interfaces that make use of those control signals and describes the materials and methods employed in the experiments. Section 4 shows the obtained results. Finally, Section 5 analyzes these results and Section 6 presents the most relevant conclusions of this work.

## 2. Previous Related Works

We review in this section the previous works related to systems based on P300 EP and on EOG.

### 2.1. P300-Based Systems

A large number of works can be found in the literature from recent years proposing the use of P300 for building assistance systems that facilitate communication and environmental control in patients with severe motor and neurological damages [1]. For instance, the P300 speller proposed by Donchin and Farwell [6] is a well-known BCI for communication purposes. It displays a virtual keyboard organized in a 6×6 matrix whose cells contain the letters of the alphabet and 1-word commands for system control. The user must focus his/her attention on one cell while rows and columns of the matrix flash alternately. EEG activity is analyzed after each flash, so the cell of interest is identified as the cell at the intersection of the row and column that elicits the largest P300 potential. The developed system achieved a spelling rate of 2 characters per minute.

Following this approach, several works have been presented that improve the system performance. For example, different configurations of the characters were proposed to avoid perceptual error in the detection of P300 produced by adjacent rows/columns. In this regard, a region-based paradigm is proposed in [26], where letters are organized into different flashing regions in the computer screen. Towsend et al. [27] also propose a change of paradigm, using a checkerboard instead of traditional row and column schemes. This novel approach outperforms the row/column paradigm in terms of accuracy and transmission rates.

Moreover, several preprocessing techniques were assessed in order to improve the P300 detection [28]. Xu et al. [29] proposed an algorithm based on Independent Component Analysis (ICA) for EEG decomposition and a signal reconstruction according to spatio-temporal patterns that enhance the P300 peak. They achieved a 100% accuracy for the dataset IIb of BCI Competition 2003. Donchin et al. [30] assessed a P300-based BCI on ten subjects employing the Discrete Wavelet Transform (DWT) with Daubechies wavelets and four levels of decomposition. Their results indicate that, using a bootstrapping approach, an offline version of the system can achieve a communication rate of 7.8 characters per minute with an 80% accuracy. Several classification algorithms have been also applied for P300 detection, such as step-wise discriminant analysis [30], Linear Discriminant Analysis (LDA) [20] or Support-Vector Machine (SVM) [16,31] among others [28,32].

Although the original P300 Speller proposed by Donchin and Farwell [6] considered only one input channel, it was discovered that the use of multiple channels improved the classification accuracy [33]. Therefore, most modern spellers employ several recording electrodes. However, a larger number of channels requires complex and expensive EEG recording devices, where each electrode must be individually placed and calibrated. Such conditions represent a limitation for home users and daily BCIs. Thus, several works using single-channel P300 systems have been recently developed. Xie et al. [23] have proposed a single-channel single-trial P300 detection based on a new method, known as Extreme Learning Machine (ELM). Their algorithm is tested on eight subjects and an average accuracy above 85% is obtained. In [22] a single-channel and single-trial P300 detection algorithm is also proposed. In this work, both DWT and ICA algorithms are combined to extract features from the II dataset of the BCI Competition III, which is based on the P300 speller paradigm. Their results show an average accuracy of 65% in single-trial P300 detection.

On the other hand, the matrix-based paradigm for stimuli presentation, introduced by the speller, have been widely applied in P300-based BCIs for environmental control [16]. In this case, the letters of the alphabet and the 1-word commands of the speller are replaced by icons associated to a control function for devices in the user’s environment. Thus, instead of selecting a character to be written, the user chooses an action to be executed in the selected device. For example, Carabalona et al. proposed a P300-based BCI for disabled people in a real smart home environment [18,19]. In this study, they presented a 6×6 matrix with icons corresponding to the smart home devices and compared its performance with the standard-character speller. Their results reported lower accuracy for the icon tasks than for the character ones, possibly due to cognitive effort when using icons. Aloise et al. [17] proposed an asynchronous P300-based BCI for home device control such as DVD players, electric lights, phone calls, and so forth, which were presented in a 4×4 matrix. They introduced a threshold-based classification approach that allowed the interface to understand the user intent when he/she is engaged in another task or is distracted by the surrounding events, avoiding false positive selections. In a more recent study, Corralejo et al. [34] have proposed an assistance tool for operating of electronic devices at home through a P300-based BCI. The interface is tested in a real scenario with fifteen severely impaired subjects which could manage eight real devices by means of 113 control commands. Out of the fifteen subjects in the study, ten achieved an accuracy higher than 75%, and eight an accuracy above 95%. Schettini et al. [35] presented a P300-based BCI for Amyotrophic Lateral Sclerosis (ALS) patients to manage communication and environmental control applications. The results showed that the BCI could be used as an assistance technology with no significant reduction of usability compared to other communication interfaces such as screens or buttons. In a recent work, Kim et al. [16] developed an online BCI for home appliances control such as TVs, electric lights or digital lock systems. They proposed to present the stimuli employing an User Interface (UI) that displays a control icon and a real time image of the corresponding appliances to jointly verify if the proposed UI works correctly in a P300-based BCI even with introduced distractions due to live image of these appliances. For this purpose, the P300 and N200 potentials were analyzed to overcome visual distractions. The results showed that the healthy participants could control the appliances via BCIs with an average accuracy ranging from 78.7% to 83%. Moreover, different stimuli configuration and flashing methods have been also applied in the P300-based BCI for environmental control. For instance, Hoffman et al. [20] presented a BCI where home appliances images flashed one at a time to elicit the P300 potential. Aydin et al. [21] proposed an internet-based asynchronous P300 BCI for environmental control applying a region-based paradigm instead of using the standard row/column one.

### 2.2. EOG-Based Systems

Several HMIs based on EOG and eye movements have been proposed in recent years. EOG signals are particularly useful for creating HMIs since they present consistent patterns with relatively large potentials, becoming easier to detect than EEG activity [9,36]. In this regard, He et al. [10] presented a single-channel EOG-based HMI that allows users to spell by only blinking. Forty characters were displayed to subjects, which were randomly flashed. In order to select one of them, the subject should blink as the target character was flashed. The results showed that eight healthy subjects achieved an average accuracy of 94.4% when selecting a character in synchronous mode and of 93.43% in asynchronous mode. Deng et al. [37] proposed a multi-purpose EOG-based HMI where different eye movements (horizontal and vertical) were detected by the system to control, for example, a TV for channel shifting or volume adjusting. The authors claim that the system can achieve above 90% of accuracy after adjustment. Also employing eye-movements and the generated EOG signal, Barea et al. [38] designed a system for controlling and guiding an electric wheelchair. The system allows users to adjust the direction and speed of the wheelchair only from eye-movement. The study successfully showed results with reduced learning and training times. In a recent study, Guo et al. [24] presented a single-channel HMI that recorded EOG activity using a self-designed patchable sensor. The captured eye-movements were converted to computer commands, including scroll up, scroll down, and close. Eight subjects were trained to use this system and became capable of making continuous control with an average accuracy of 84%. Heo et al. [39] also designed a wearable forehead EOG measurement system for HMIs. In this case, vertical and horizontal movements of the eye were detected. The system was tested for three applications: a virtual keyboard using either a modified Bremen BCI speller or an automatic sequential row/column scanner, and a drivable power wheelchair. The results showed a typing speed of 10.81 and 7.74 letters per minute and an accuracy of 91.25% and 95.12% for the BCI speller and the row-column scanner, respectively. For the wheelchair experiment, the user drove through an eight-shape course without collision with obstacles.

EOG control signals have been combined with EEG control signals in order to build more robust and reliable hybrid BCIs. A hybrid BCI is a mixed approach where the technology of conventional BCIs is combined with another system in order to improve its performance [9,40]. In this context, different hybrid systems have been proposed combining EEG and EOG signals [41], such as SSVEP and blink-related EOG signals [9], MI tasks and blinking EOG activity [42] and also the P300 EP combined with ocular movements [43,44].

Thus, P300- and EOG-based BCIs have been very active research fields during the last decades, and numerous successful works have been presented. However, clinical devices are usually employed in these works for the capture of the user’s brain or ocular activity. The main drawback of these devices is that they typically consist of a large number of sensors and are very expensive, which represents a limitation to build a daily-life BCI accessible for the general public. In order to overcome these limitations, in this study we propose a low-cost single-channel BCI based on a consumer-grade EEG device and available to be used without expert knowledge.

## 3. Material and Methods

**Figure 1:**
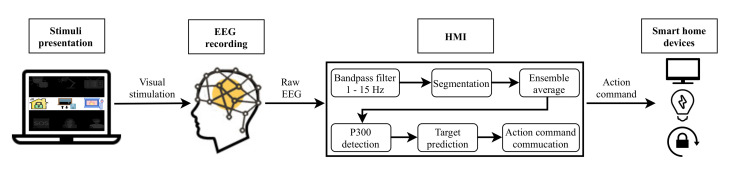
Electroencephalography (EEG) system based on P300 EP.

**Figure 2:**
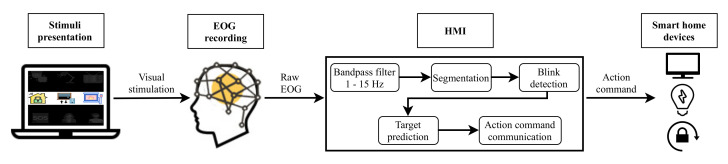
Electrooculography (EOG) system based on eye blinks.

Two different systems have been developed in this study. Figure 1 depicts the architecture of the EEG control system based on P300 EP. Figure 2 shows the EOG system based on eye blinks. Both systems are made up of four main parts: a GUI for stimuli presentation, a hardware device for EEG/EOG signal recording, the HMI for analyzing the captured signal and converting it into an action command and, finally, household devices for the execution of actions. The following sections describe each of these components.

### 3.1. Graphical User Interface

As depicted in Figure 1 and Figure 2, for both developed systems, the objects that the user can interact with are presented on a laptop screen using a GUI. This stimulation program was designed and developed in Python employing the PsychoPy package [45].

**Figure 3:**
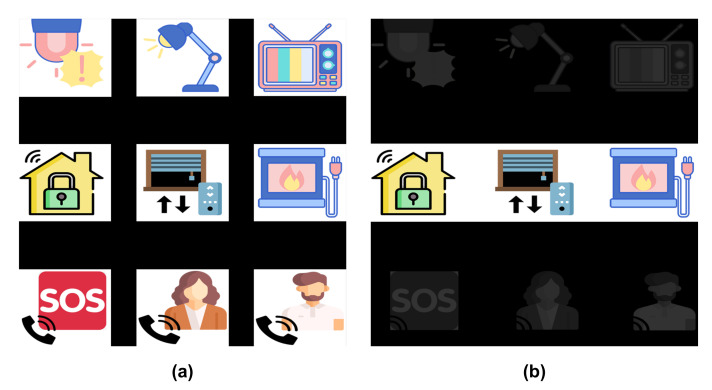
Graphical User Interface (GUI) for the two stimulation paradigms. (**a**) represents the 1-by-1 stimulation paradigm with all the elements intensified, while (**b**) corresponds to the row/column stimulation paradigm only with the second row intensified.

Figure 3 shows an example of the GUI, composed by a 3×3 matrix containing nine images describing the objects and control functions to be executed by users. The first two rows correspond to home devices, such as TVs, digital house locks or electric lights, which have a state that can be switched (e.g., on/off or up/down) by selecting it with the HMI. The last row has three possible phone calls: the emergency call (i.e., an SOS number) and two favourite contacts in their phone-book.

Two stimulation paradigms were assessed for the experiments. For the first paradigm, each element of the matrix is intensified one by one. This intensification is randomly performed, but all the elements are intensified the same number of times. Therefore, they will have the same probability of being intensified, that is, p=1/9. Moreover, an element cannot be intensified two consecutive times.

For the second paradigm, instead of element by element, each row and column of the matrix is intensified. This is also done randomly, and they are intensified the same number of times. Therefore, the probability of one element being intensified at any given time is p=2/6=1/3.

Figure 3 shows both stimulation paradigms: on the left side, the 1-by-1 paradigm with all the elements intensified and, on the right side, the row/column paradigm with the second row intensified.

**Figure 4:**
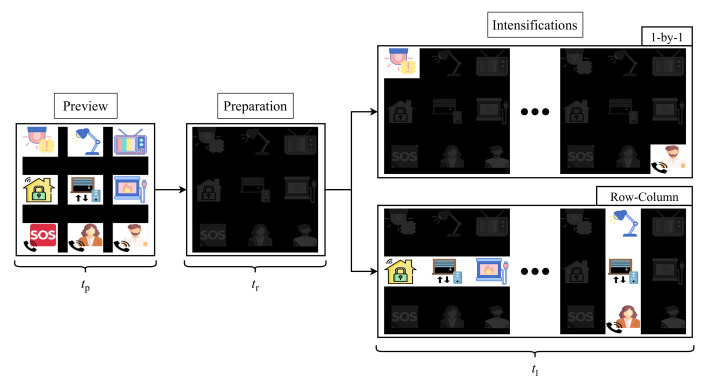
Sequence of steps in the two stimulation paradigms.

The developed application for stimuli presentation is configurable and it can be adapted to the user’s requirements. However, in order to obtain comparable results, we have selected the same parameters for all the participants of the study. Figure 4 shows the stimulation procedure for one run of the experiment. Each run starts with a preview period of $t_{p}\;\mathrm{seconds}$ for all the elements of the matrix, so the user can locate its target before the intensifications begin. Following this first step, a dark matrix is shown to the user during $t_{r}\;\mathrm{seconds}$. At this stage, the target must be located and the user must focus his/her gaze on it for the rest of the run. Once this preparation ends, the intesification step starts. The duration of this stage is conditioned by the selected stimulation paradigm (1-by-1 or row/column) and by the recording mode (EEG or EOG), since the number of intensifications and times will vary for each of them. Remember that these intensifications are randomly performed that is, the user does not know the order in which the object or column/row will be intensified. These parameters are explained in detail in the following sections.

### 3.2. Hardware

EEG and EOG data have been recorded using the OpenBCI Cyton Board with a sampling frequency of 250Hz. The Cyton board allows simultaneous recording from eight channels, but only one of them is employed in this study. Two types of electrodes can be employed for capturing the EEG and EOG activity of the user—wet and dry electrodes. Traditionally, wet electrodes have been employed for biopotential recordings [46]. This procedure implies skin preparation and the use of wet gels or saline liquid to create a conductive path that improves signal quality and reduces skin impedance. However, this gel will eventually dry out, resulting in a poor quality signal and the need for electrode replacement. Moreover, for high-density electrode montages, the procedure can become cumbersome and uncomfortable for the user. To overcome these issues, dry electrodes that do not require any gel application [47,48] or semi-dry electrodes that only employ a tiny amount of conductive gel [49,50,51] have been proposed and implemented in HMIs in recent years. However, although these new dry electrodes offer a faster setup time and greater user comfort, they usually present a higher skin impedance than the wet ones [52,53,54]. Since this study aims to analyze the control signals on each user and determine which one offers the best performance, we decided to employ wet electrodes to obtain high-quality and low-impedance signals.

**Figure 5:**
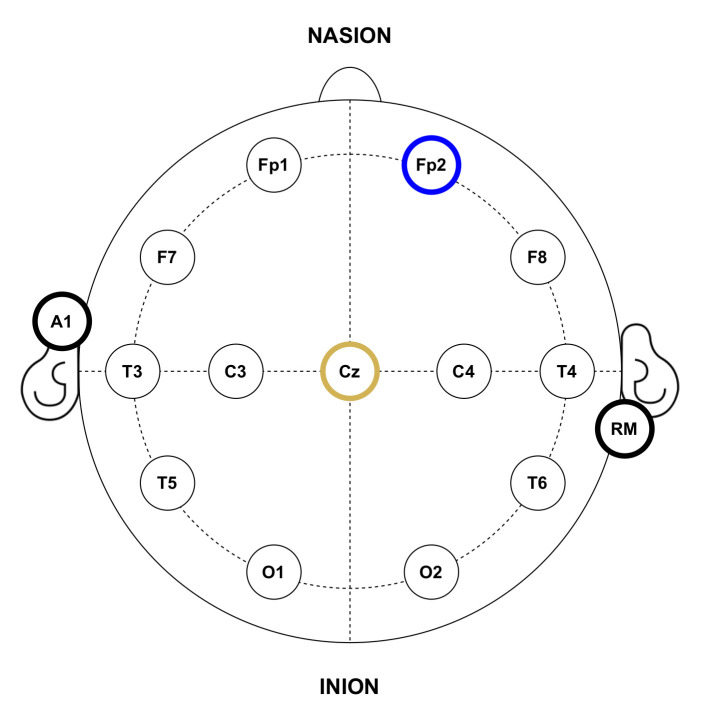
Anatomical electrode distribution in accordance with the standard 10–20 placement system used during the experiments. The yellow circle represents the input channel for EEG recordings, while the blue one corresponds to EOG recordings. Black bordered circles represent reference and ground electrodes.

In the experiments, gold cup electrodes were placed in accordance with the 10–20 international system for electrode placement [55] and attached to the subjects’ scalp using a conductive paste. The electrode-skin impedance was checked to be below 15kΩ at all the electrodes. Following this placement system, each electrode location is identified by numbers, the even ones representing the right hemisphere and the odd ones the left hemisphere; and letters, corresponding to the lobe and area of the brain where they are placed: pre-frontal (FP), frontal (F), temporal (T), central (C), parietal (P) and occipital (O). For EEG recordings, since the objective is to detect the P300 potential, the electrode for the input channel was located in the Cz position. Conversely, for EOG recordings, the electrode was placed at Fp2 in order to detect activity related to eye blinks. Both recording modes shared reference and ground electrodes, placed at the right mastoid (RM) and the left earlobe (A1), respectively. It is important to note that this electrode location will depend on the purpose of the HMI and the control signals it wants to capture, for example, a BCI that wants to detect the eye state of the user (closed or open eyes) would probably place the input electrodes on the occipital area of the brain where the visual cortex is located [25]. Figure 5 shows the electrode placement for the experiments.

### 3.3. EEG Recording and Signal Processing

Classical electrode location for P300-based EEG applications generally contains three typical positions: Fz, Cz and Pz [56,57], since the P300 potential is maximally recorded from the midline centroparietal regions [58]. Therefore, the only channel used by the proposed EEG system shown in Figure 1 is placed at Cz position. The input signal is filtered using a 16th-order Butterworth infinite response filter between 1 and 15Hz.

Evoked potentials appear as event-related responses from the brain to sensory stimulation, such as the one used in this study (visual stimulation). The main challenge to overcome when working with these brain responses is that individual EPs present very low amplitude values, ranging from 0.1 to 10μV, whereas the EEG background activity ranges from 10 to 100μV, which causes EPs to be hidden among the background information. Fortunately, EPs are time-locked to stimuli that is, they usually occur with the same latency from the stimulus onset. Conversely, background EEG not related to the stimulus will fluctuate randomly. A widely applied technique that takes advantage of this effect is ensemble averaging, which is based on averaging all the brain responses related to stimuli presentation. In this procedure, the activity time-locked to stimuli onset will remain robust, while the random background EEG will not, so the Event-Related Potentials (ERPs) will appear clearly and the noise will be cancelled [59,60].

Consequently, we chose this processing technique for the acquisition of the ERPs and detection of P300 EPs. In order to apply it, the EEG signal is first segmented into epochs that are time-locked to stimuli. Following the criteria introduced by Farewell and Donchin in the original P300-speller [6], which states that the useful data consisted of the recorded EEG for 600ms after the onset of each intensification, epochs of this duration are extracted after each stimulus presentation. Once all the epochs have been extracted, an ensemble average, that is, the average of all the epochs for each stimulus, is calculated. The recorded EEG signal, x, can thus be segmented according to the *k* stimulus to obtain an ensemble of *M* epochs, which in the discrete domain would be represented by *N* samples.

(1) $$x_{k,i}\left(n\right),\;i=1,\ldots ,M;\;n=0,\ldots ,N-1;\;k=1,\ldots ,K.$$

The ensemble of epochs for the stimulus *k* can be represented in a matrix form, $X_{k}$, where each row vector represents the different epochs and each column vector the samples of the recorded EEG.

(2) $$X_{k}=\left[\begin{array}{ccc} x_{k,1}\left(0\right) & \cdots & x_{k,1}\left(N-1\right)\\ \vdots & \ddots & \vdots \\ x_{k,M}\left(0\right) & \cdots & x_{k,M}\left(N-1\right) \end{array}\right].$$

The size of the resulting matrix will be N×M, where each element $x_{k,i}\left(n\right)$ represents the nth sample of the ith epoch for the kth stimulus.

The ensemble average for each stimulus, $s_{k}$, can then be easily calculated by averaging all its epochs as follows

(3) $$s_{k}=\frac{x_{k,1}+x_{k,2}+\cdots +x_{k,M}}{M}.$$

Once the ensemble average for each stimulus has been obtained, the P300 potential should clearly appear in the resulting averaged signal for the target stimulus. Therefore, if the HMI is able to automatically detect this P300, it will predict the user’s will without any physical intervention. Several methods have been proposed for detecting the P300 potential [61]. In this study, we define a P300 window that contains and surrounds the potential, so the peak and area of the averaged signal in this window can be used for prediction [29]. The elements with the highest area inside the P300 window will be considered as possible targets.

In order to detect the target elements, one prediction criteria was applied for each stimulation paradigm. On the one hand, for the 1-by-1 paradigm, the element with the highest area inside the P300 window was selected as target. However, for the row/column paradigm, the row and column with highest area were selected and the target element was identified as the element in their intersection.

### 3.4. EOG Recording and Signal Processing

For the EOG system shown in Figure 2, data are captured employing only one input channel placed on the forehead at the Fp2 position. This electrode location is selected due to the blinking activity being concentrated at the frontal regions [9]. Then, data is filtered using a 4th-order Butterworth infinite response filter between 1 and 15Hz.

Users must blink when their target element is intensified. The purpose of this action is to communicate the objective element to the HMI so it can execute its associated action. This voluntary blink is time-locked to stimuli, since it must appear right after the intensification onset of the target element and before the onset of the next intensification. Taking this into account, the recorded EOG signal can be segmented into epochs of 1s according to each intensification. An epochs’ ensemble for the stimulus *k* can be represented in a matrix form, $X_{k}$, as in (2), where each row vector of the matrix represents the epoch for stimulus *k* and each column vector represents the samples of the recorded EOG signal. Hence, the element $x_{k,i}\left(n\right)$ corresponds to the nth sample of the ith epoch for the kth stimulus.

Since EOG activity and, especially, blinking movements present consistent patterns with large potentials, they are easier to detect than EEG brain responses [9,36] and, therefore, there is no need to present the stimuli as times as with EEG in order to reduce background noise.

Once the recorded signal has been segmented, the HMI must detect the blinks in the extracted epochs in order to predict the user’s will and execute the desired action. Fortunately, blink movements present clear and characteristic patterns in EOG signals with two consecutive large peaks, positive and negative, respectively, corresponding to the closing and opening of eyes. These large peaks can be easily recognized from background EEG activity, which presents smaller amplitude values [8,62]. Consequently, a threshold-based detection algorithm is proposed in this study. Two specific threshold values are defined: $th_{n}$, for the negative peak and, $th_{p}$, for the positive. Each epoch is analyzed and a resulting vector v is constructed, where its nth sample follows this criteria,

(4) $$v_{k,i}\left(n\right)=\left\{\begin{array}{cc} -1, & x_{k,i}\left(n\right)<\;th_{n},\\ \;1, & x_{k,i}\left(n\right)>\;th_{p},\\ \;0, & th_{n}\leq x_{k,i}\left(n\right)\leq th_{p}, \end{array}\right.$$

which represents the threshold vector for the ith epoch of the kth stimulus.

According to (4), the observed epochs can be divided into blinking and non-blinking intervals. Blinking intervals will be those between $v_{k,i}\left(n\right)=-1$ and $v_{k,i}\left(n\right)=1$ that is, between the closing and opening of eyes, while non-blinking intervals will be those outside these values. The duration for blinking movements ranges from 100 to 400ms [8,63], so blinking intervals longer than 400ms are discarded. Thus, it will be considered that a blink is produced in a specific epoch when it contains a valid blinking interval that is, the closing and opening of eyes have been detected in the lapse of 400ms. The BCI can predict the target element by counting the blinks produced on each of its epochs. Depending on the stimulation paradigms, two prediction criteria were applied: for the 1-by-1 paradigm, the element with the same number of blinks as experiment runs was considered the target; for the row/column paradigm, the row and column with the same number of blinks as experiment runs were selected and the target was determined to be the element on their intersection.

### 3.5. Experimental Procedure

The participant group in our experiments included a total of nine volunteers who agreed to collaborate in this research. The participants indicated that they did not have hearing or visual impairments. Informed consents were obtained from all the participants in order to employ their data in our study. Table 1 summarizes the information of each subject that took part in the experiments.

**Figure 6:**
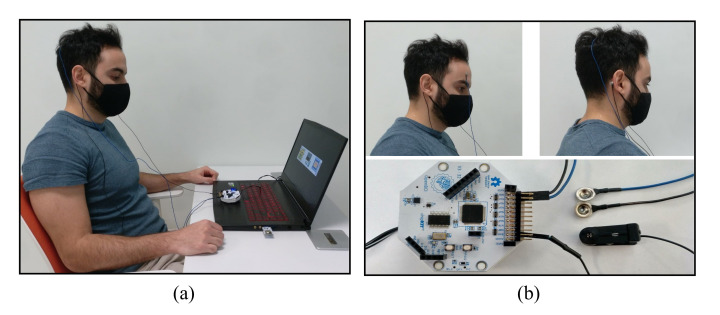
Recording session from one participant of the study, electrode placement for the experiments and recording device: (**a**) P300 recording session; (**b**) electrode locations for blinking experiments depicted on the upper left corner; electrode locations for P300 experiments depicted on the upper right corner, and the Cyton board employed for recording the data shown on the bottom.

The experiments were carried out in a sound-attenuated room where the participants were invited to sit in a comfortable chair while focusing their attention on a 15.6-inch laptop screen where stimuli were presented. Figure 6 shows a participant during the P300 recording session, the electrode placement for both experiments and the recording device.

Recording sessions for each participant were divided into four independent experiments, one per each stimulation paradigm and recording mode that is: for the 1-by-1 paradigm with EEG signals, for the row/column paradigm with EEG signals, for the 1-by-1 paradigm with EOG signals and for the row/column paradigm with EOG data.

As shown in Figure 4, each experiment was divided into runs consisting of three stages: preview, preparation and intensifications. The GUI described in Section 3.1 was configured with $t_{p}=5\;s$ and $t_{r}=2\;s$. The duration of the intensification stage, $t_{I}$, was conditioned by four different parameters, as follows,

- Number of Stimuli (NS): nine for the 1-by-1 paradigm (one per each element of the matrix) and six for the row/column paradigm (one per each row and column of the matrix).
- Number of Instensifications (NI) that is, the number of times that each stimulus is presented to users.
- Intensification Time (IT) that is, the time that a stimulus remains intensified.
- Inter-Stimuli Interval (ISI), the elapsed time since the stimulus intensification ends to the onset of the next one. During an ISI, any element is intensified: all of them stay dark.

Consequently, $t_{I}=\mathrm{NS}\times \mathrm{NI}\times \left(\mathrm{IT}+\mathrm{ISI}\right)$. Table 2 summarizes the different configurations selected for each experiment type. The same parameters were used for all the participants in the study.

After each run, a rest interval of at least 30s was allowed. The entire experimental session lasted approximately 90min. As the analysis of the collected data is performed offline, no visual feedback is provided to the users when each run ends.

## 4. Experimental Results

### 4.1. Signal Analysis

Captured EEG signals from all the participants in this study were analyzed and visually inspected in order to verify that their recordings were correctly performed and that the P300 EP was elicited when the target element was intensified. As previously described, the EEG signal was filtered, epoched and averaged for this purpose.

**Figure 7:**
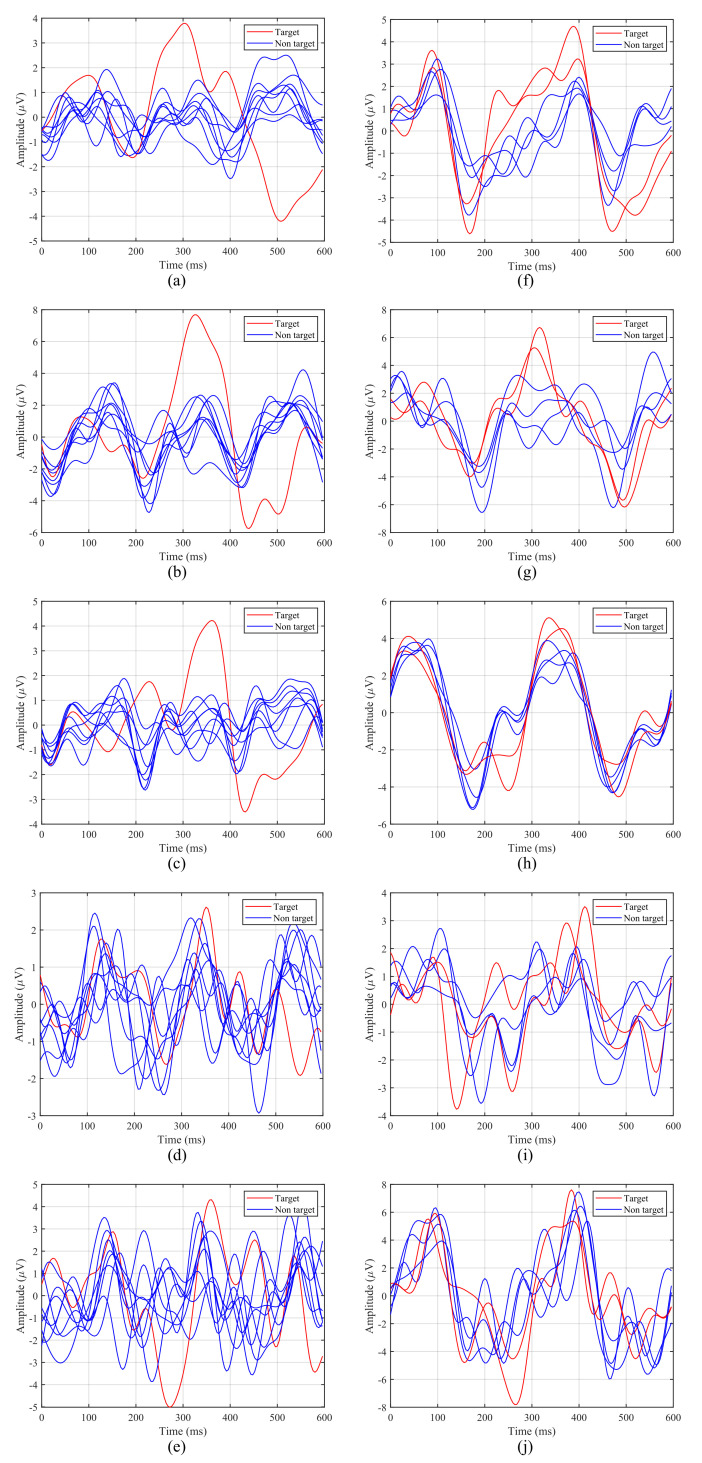
Averaged Event-Related Potentials (ERPs) from the first five participants for both stimulation paradigms: (**a**–**e**) 1-by-1 paradigm; (**f**–**j**) row/column paradigm.

Figure 7 shows the averaged ERPs for the first 5 participants with both intensification paradigms considering only one run of our experiment. The left column contains ERPs for the 1-by-1 paradigm, while the right one corresponds to the row/column paradigm.

The P300 potential appears clearly around 300ms for Subjects 1, 2 and 3 when the target element is intensified, whereas for Subjects 4 and 5, although the potential appears, it is more difficult to distinguish. In addition, it can be observed that the latency, that is, the elapsed time from the stimulus onset to the highest value of the P300 potential curve, varies from one participant to another. For example, for the 1-by-1 paradigm, the first subject (Figure 7a,f) reaches its highest value at 304ms, whereas for the third subject (Figure 7c,h) this happens at 360ms. For the first subject the delay also varies for each paradigm, since for the row/column one the peak appears closer to 400ms (Figure 7f).

Taking this analysis into account, the P300 window, defined by the BCI for the P300 detection and the subsequent target identification, will depend on the latency of the participants for each paradigm. Therefore, the BCI must define a P300 window adapted to each participant and condition, since a general common window would offer poor results.

**Figure 8:**
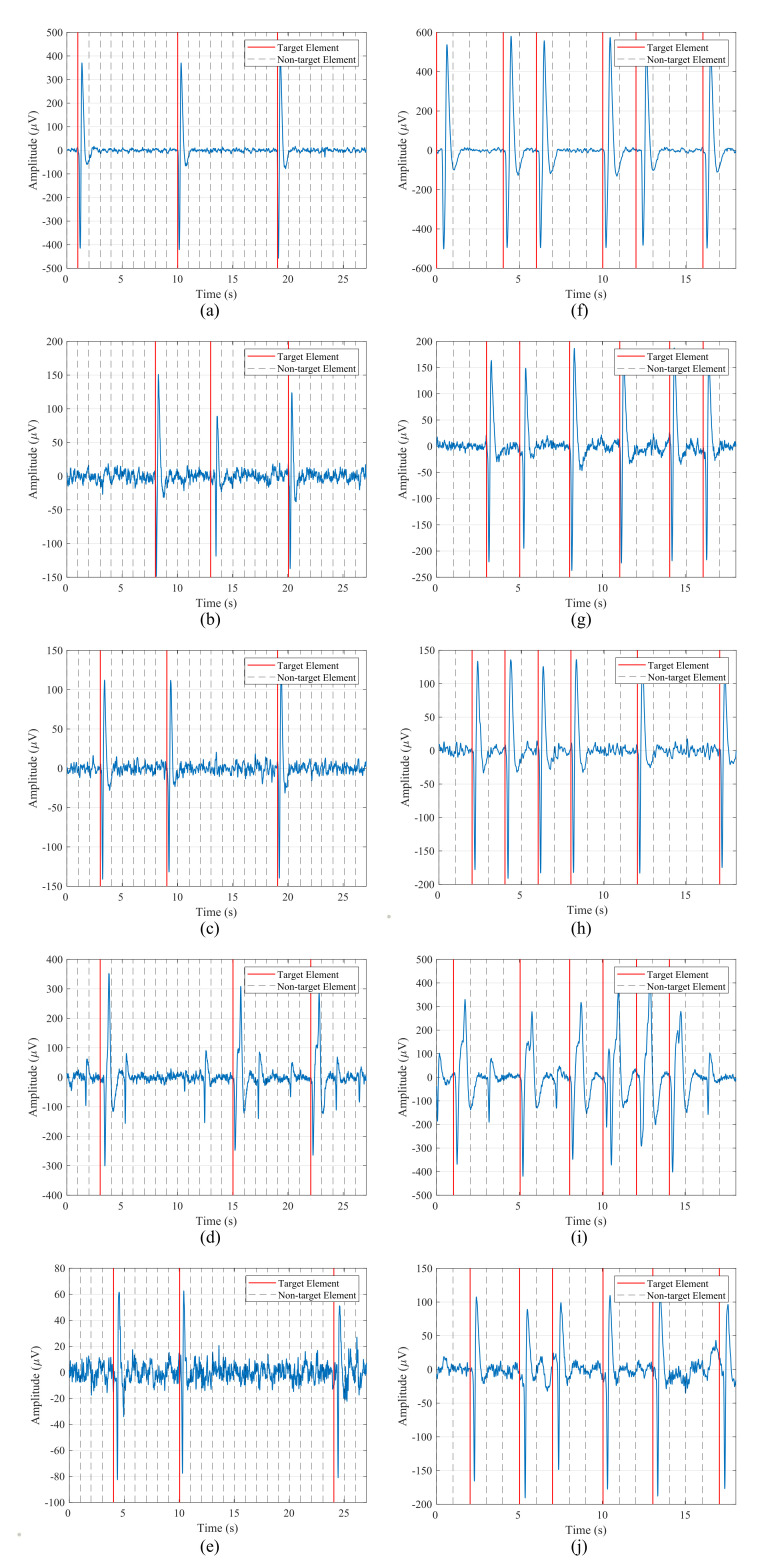
Data from the first five participants for one run of the blinking experiment for both stimulation paradigms: (**a**–**e**) 1-by-1 paradigm; (**f**–**j**) row/column paradigm.

EOG recordings were also analyzed and visually inspected in order to verify that the data were correctly captured and that the blinks were well represented in the signals. Figure 8 shows data from the first five participants for one run of the blinking experiment and both stimulation paradigms. The left column contains the signal for the 1-by-1 paradigm, while the right one corresponds to the row/column one. The blinks, which are represented by two consecutive large peaks, negative and positive, are clearly distinguishable from the background EEG data captured by the electrode. These voluntary blinks appear right after the intensification onset of the target element, so the user is communicating to the HMI the element that he/she wants to select. It is important to note that the amplitude of these blinks varies for each participant. For example, for the first subject, blink peaks are above 300μV, while for the second subject they appear from 80 to 150μV.

Taking this amplitude variation into account, the threshold value employed by the blink detection algorithm must be subject-dependent. That is, the BCI must define a threshold value for each participant in order to detect the produced blinks in the epochs.

### 4.2. Classification

High classification accuracy when detecting the element selected by the users is of primary importance for the correct performance of the HMI. Low accuracy would imply the execution of the wrong action in the user’s environment, which could be frustrating, annoying and, in some cases, it could even mean a risk for him/her, for example, if the user needs to call emergency services but the interface does not detect it correctly. Consequently, the accuracy of the classification algorithms presented in this study is assessed and analyzed in order to determine if their results are suitable for the implementation of a reliable HMI with practical applications in a smart-home environment.

Table 3 shows the classification accuracy for each subject, stimulation paradigm and both control signals. This accuracy is calculated according to the number of runs that were correctly classified by the HMI. An accuracy of 100% is achieved when the interface is able to correctly predict each target element in the eight runs of the experiment.

For the P300 experiments, the 1-by-1 stimulation paradigm presents significantly higher results than the row/column one, with all the participants above 75% and five of them reaching 100% of accuracy. Conversely, the row/column paradigm offers very poor results, with five out of the nine subjects below 63%. In the blinking case, both paradigms achieve similar results, offering high classification rates with comparable average accuracy.

### 4.3. Response Time

The response time of the HMI is also an important feature to be evaluated. Long response times will produce fatigue, frustration and anxiety in the users, which can lead to a bad performance of the interface and a loss of interest in the system. We must find the shortest response time for the HMI that does not compromise the classification accuracy. Thus, the time response of each stimulation paradigm and control signal was assessed.

**Figure 9:**
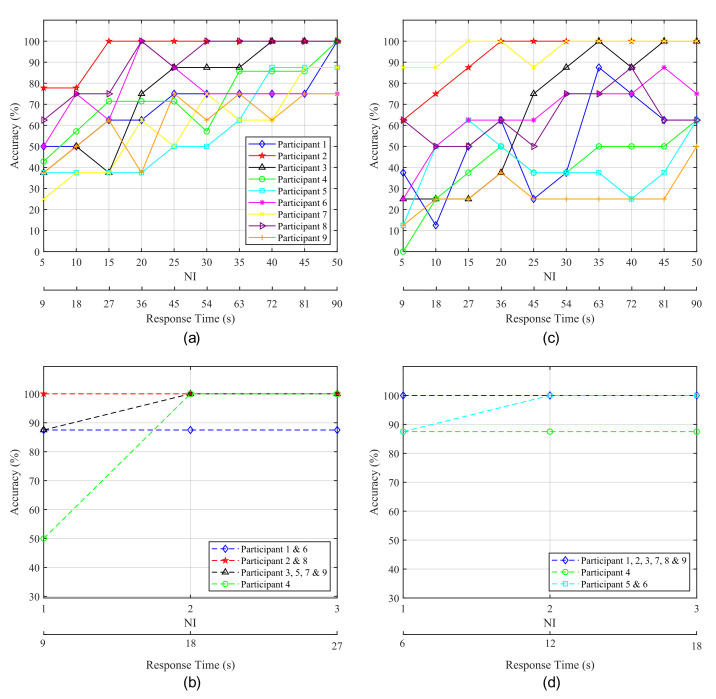
Accuracy obtained for each participant as a function of the elapsed time from the start of the intensifications. (**a**,**c**) correspond to P300-based experiments while (**b**,**d**) correspond to blink-based experiments. The left column (**a**,**b**) shows the results for the 1-by-1 paradigm and the right one (**c**,**d**) depicts the results for the row/column paradigm.

Figure 9 shows the average classification accuracy obtained for each participant as a function of the number of intensifications performed for each stimulus, which is directly related to response time. Figure 9a,c show the accuracy obtained from the P300 experiments. Figure 9a shows the results obtained from the 1-by-1 paradigm while Figure 9c shows those from the row/column paradigm. It is apparent that there exists a trade-off between NI and accuracy of the HMI that is, as NI increases the obtained accuracy improves, and vice versa. This is due to the fact that a higher number of intensifications implies a larger number of epochs for the ensemble average and, therefore, the P300 becomes easier to detect. As a consequence, a higher classification accuracy means longer response times.

From Figure 9a it can be observed that, for the 1-by-1 paradigm, Subjects 3 and 5 achieved their highest accuracy with 40NI at 72s. A significant improvement can be seen for Subject 2, who reaches 100% accuracy at 27s with only fifteen intensifications, and for Subject 8, who achieves a stable 100% accuracy at 30 NI. Subject 6 also achieves a stable accuracy with 30 intensifications, but with poorer results. Conversely, for Subjects 1, 4 and 9, all or almost all the possible intensifications are needed to achieve a high and stable performance. In the case of the row/column paradigm (Figure 9c, lower results are obtained and more time is required to achieve the best performance. Subject 7 is the only one that shows an improvement over the 1-by-1 paradigm. Subject 2 exhibits excellent results, but slower response time than for the 1-by-1 paradigm. Subject 3 also reaches a good accuracy, but the other three subjects do not show a stable behaviour, since their performance drop even when the elapsed time and the number of intensifications increase. Therefore, it becomes difficult to determine which specific response time offers the best accuracy.

Blinking experiments can be also analyzed in order to evaluate the response time of the system. In this case, the delay in the response is also dependent on the number of intensifications performed for each stimulus. It should be noted that, for these experiments, every time the target stimulus is presented, the user must blink in order to communicate its selection to the HMI. Therefore, the higher the NI, the larger the response time and the number of blinks required to select the target element. We must take into account that if a very low number of blinks is employed for this purpose, the false positive rate will increase, since non-voluntary blinks can be misinterpreted as control signals sent by users. If the number of blinks needed to pick the target is higher (e.g., two or three blinks), the interface can employ a double or triple verification system and non-voluntary blinks will probably not be interpreted as voluntary control signals. Therefore, for EOG there also exists a trade-off between response time and reliability, since a large number of blinks for target selection implies a longer response time and a lower false positive rate.

Figure 9b,d show the accuracy of the HMI obtained for EOG experiments as a function of NI and the response time. Figure 9b shows the results for the 1-by-1 paradigm and Figure 9d those corresponding to the row/column paradigm. For both cases, some subjects achieve their highest accuracy with one and two intensifications and no improvement is produced when using the maximum NI. However, it can be observed that for one intensification (i.e., only 1 blink for target selection), several subjects show a lower accuracy due to non-voluntary blinks mistaken as control signals. Therefore, two intensifications seem the most suitable option, since mistakes produced by non-voluntary blinks can be avoided and the response time of the system is kept low.

## 5. Discussion

In this study, we have developed an HMI for environmental control using a single-channel recording system. The P300 potential and eye blinks are compared as control signals in order to determine which one offers the best performance in terms of accuracy and response time. The home elements to be controlled are displayed in a GUI following a matrix-form and presented to users using two different stimulation paradigms: (1) home elements are intensified one by one or (2) all the elements of the same row/column are jointly intensified at the same time. Both interfaces, either the P300-based interface or the blink-based interface, employ only one input channel of the same EEG device to capture the brain/eye user’s activity. In addition, the GUI to show the stimuli is fully configurable and able to implement both paradigms. Thus, both interfaces exhibit similar cost and complexity for their respective implementations in real environments.

Practical and real applications of the HMIs will differ among users according to their degree of muscular control. The P300-based HMI does not require voluntary muscle activation for controlling and communicating with external devices. Thus, their immediate users will be those who suffer a complete locked-in state with a loss of all motor control or whose remaining control is easily fatigued or otherwise unreliable. This user group includes totally paralyzed patients due to, for example, terminal stages of ALS or brainstem stroke, or users suffering from movement disorders that abolish motor control caused by, for example, cerebral palsy [1]. For these patients, even the most simple HMI for turning on/off a home device is a valuable tool [7]. However, most potential users have better conventional options for communication. For example, those who retain control of only a single muscle, such as the eyelids, can use it to send control signals in a faster and more accurate way than that provided by EEG-based HMIs [7]. The results presented in this study agree with this statement, as shown in Table 3 and Figure 9, where it is apparent that the blink-based interface offers shorter response times and similar or higher accuracy in classification than the P300-based HMI.

The subjects of this study, all able-bodied with no motor disabilities, indicated their preference for the blink-based interface since it is not as mental-demanding and time-consuming as the P300-based interface. Moreover, they also pointed out they felt with greater control over the system when using blinks as control signals, by marking the target element with voluntary and conscious actions.

From the EEG recordings, depicted in Figure 7, it is apparent that the latency of the P300 potential varies for each subject. As has been already studied [64], individual differences on the P300 latency are related to mental function speeds and cognitive capabilities, such that a shorter latency corresponds to a higher cognitive performance [65,66]. Moreover, the P300 latency is reduced with children and increases with normal ageing [64,67,68]. It can also be observed in Figure 7 that the P300 potential appears clearly with the 1-by-1 paradigm, especially for the three first subjects, while for the row/column experiments the P300 curve generated by the target stimulus does not show big differences with respect to other stimuli. A possible explanation for this behaviour is the human perceptual error produced by targeting adjacent rows and columns [26]. According to this phenomenon, when rows/columns adjacent to desired elements are intensified, they also elicit a P300 potential, which can be confused with the one elicited by the target element. As a consequence, as shown in Table 3, the row/column paradigm offers a classification accuracy significantly lower than that obtained by the 1-by-1 paradigm, where all the participants achieve an accuracy above 75%.

Notable differences can also be observed between both paradigms regarding time responses, shown in Figure 9, in which the 1-by-1 paradigm shows a more stable behaviour and, with 45s, five of the nine participants are above 70%. All of the subjects were above 70% for 81s.

**Figure 10:**
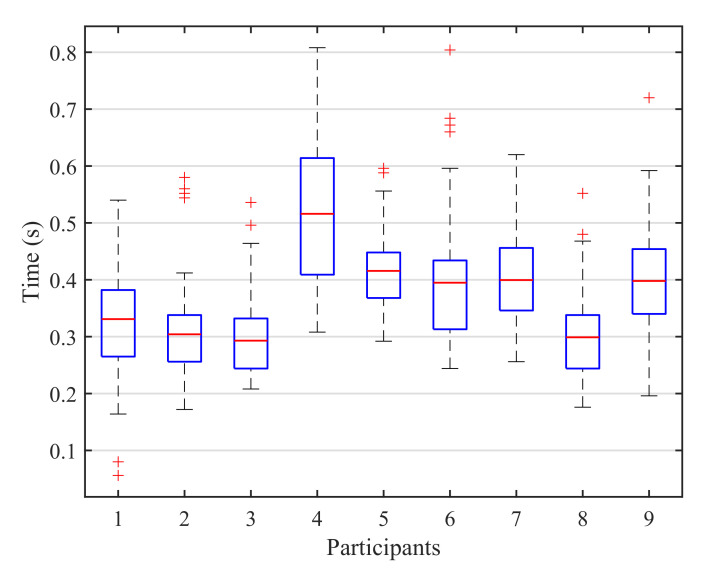
Box plot of the reaction times for all the blinks performed by the subjects across all the blinking experiments. On each box, the average response time is marked as the central red line and the bottom and top edges of the box indicate the 25th and 75th percentiles, respectively. The whiskers extend to the maximum and minimum reaction time not considered as outliers. The outliers are individually plotted using the ‘+’ symbol.

On the other hand, for the blink detection experiments, the results achieved by both stimulation paradigms are very similar, only varying for three of the subjects and with a close average accuracy of 97.22% for the 1-by-1 paradigm and 98.61% for the row/column paradigm (see Table 3). Moreover, since the number of possible stimuli for the row/column paradigm (6) is lower than for the 1-by-1 one (9), the response time for row/column will be shorter, as shown in Figure 9. This response time can be further reduced if we analyze the reaction time of each participant that is, the elapsed time from the presentation of the target element to the corresponding user blink. In our experiments, the time between the onset of the intensification of one element and the onset of the next one is fixed to 1s (see Table 2). However, if the reaction time of a particular subject is faster, the period between two consecutive intensifications can be reduced and, consequently, the final response time of the system will be shorter. Figure 10 shows a box plot of the reaction times for all the blinks performed by the subjects across all the blinking experiments. On each box, the average response time is marked as the central red line and the bottom and top edges of the box indicate the 25th and 75th percentiles, respectively. The whiskers extend to the maximum and minimum reaction time not considered as outliers. The outliers are individually plotted using the ‘+’ symbol. The median response time for all the subjects is below 0.55s, and no blink exceeds 0.85s, so the period between two consecutive instensifications could be reduced by at least 0.15s, which would also decrease the overall response time of the system.

The proposed HMIs should be implemented in an IoT environment for smart-home control using the shortest response times while providing a high reliability of the system. Practical applications of an HMI and their implementation in real environments are strongly conditioned by their response times and accuracies [7]. Table 4 shows the shortest response time for both stimulation paradigms and control signals while keeping an average accuracy higher than 80%. Note that, for the blink-based experiments, although the results achieved by the minimum number of blinks are higher than 80%, they are not taken into account due to their sensitivity to non-voluntary blinks, which could badly influence the final performance of the HMI. Both stimulation paradigms offer a similar accuracy, but shorter response times are achieved for the row/column paradigm, so it is the most suitable option for a blink-based HMI. Conversely, for the P300 experiments, the row/column paradigm does not achieve an 80% of accuracy, so the 1-by-1 paradigm, with a response time of 63s and an accuracy of 80.36%, is the preferable option.

The proposed HMI systems can be used for non-critical applications where short response times are not required. The objective of these interfaces is to control basic functions of home devices, such as on/off switching of lights or raising/lowering window blinds. Thus, ensuring that the system performs the correct action is more important than providing a fast response.

## 6. Conclusions

We have developed an environmental control HMI using a low-cost and open-hardware recording device that captures EEG and EOG signals from one single input channel. For this purpose, eye blinks and the P300 potential are assessed and compared in order to determine which is the most suitable control signal for the HMI implementation. A fully configurable GUI has been developed for stimuli presentation and two stimulation paradigms were evaluated: the elements to be controlled are intensified one by one, or all the elements of the same row/column are jointly intensified at the same time. The obtained results show that the blink-based HMI using the row/column paradigm offered the best performance in terms of accuracy and response time. However, this interface requires voluntary muscle activation for controlling the eyelids movement, which could be a limitation for some potential users of the interface. In this case, the P300-based HMI using the 1-by1 stimulation paradigm proved to be the most reliable and suitable option. The analysis of the response times obtained by each system shows that the proposed HMIs can be used for non-critical applications where short response times are not required.

## Figures and Tables

**Table 1:** Information of participants in the experiments.

Participant	Age	Gender
1	26	Male
2	26	Female
3	26	Male
4	50	Female
5	53	Male
6	26	Male
7	26	Male
8	27	Male
9	26	Male

**Table 2:** Parameters selected for each experiment performed during recording sessions. NS: Number of Stimuli; NI: Number of Intensifications; IT: Intensification Time; ISI: Inter-Stimuli Interval; $t_{I}$: time of the intensification stage.

Experiment	Recorded Signal	Stimulation Paradigm	Runs	NS	NI	IT (ms)	ISI (ms)	$t_{I}$ (s)
1	EEG	1-by-1	8	9	50	70	130	90
2	EEG	Row/column	8	6	50	100	200	90
3	EOG	1-by-1	8	9	3	500	500	27
4	EOG	Row/column	8	6	3	500	500	18

**Table 3:** Classification accuracy (in %) for each subject, stimulation paradigm and control signal.

Participants	P300		Blink	
	1-by-1	Row/Column	1-by-1	Row/Column
1	100	62.50	87.50	100
2	100	100	100	100
3	100	100	100	100
4	100	62.50	100	87.50
5	87.50	62.50	100	100
6	75	75	87.50	100
7	87.50	100	100	100
8	100	62.50	100	100
9	75	50	100	100
Average	91.67	75	97.22	98.61

**Table 4:** Response time for both stimulation paradigms and control signals that provides an average accuracy higher than 80%. For the blink-based experiments, the minimum number of blinks are not taken into account due to their sensitivity to non-voluntary blinks.

	P300		Blink	
	1-by-1	Row/Column	1-by-1	Row/Column
Accuracy (%)	80.36	No value	97.22	98.61
Response time (s)	63	No value	18	12

## Data Availability

Supplementary material contains data registered from one participant. Other data presented in the study are available on request from the corresponding author.

## Supplementary Materials

The following are available online at https://www.mdpi.com/1424-8220/21/6/2220/s1.

## Abbreviations

The following abbreviations are used in this manuscript:

ALS	Amyotrophic Lateral Sclerosis
BCIs	Brain-Computer Interfaces
DWT	Discrete Wavelet Transform
EEG	Electroencephalography
ELM	Extreme Learning Machine
EOG	Electrooculography
ERPs	Event-Related Potentials
GUI	Graphical User Interface
HMIs	Human-Machine Interfaces
ICA	Independent Component Analysis
IoT	Internet of Things
LDA	Linear Discriminant Analysis
MI	Motor-Imagery
NS	Number of Stimuli
NI	Number of Intensifications
IT	Intensification Time
ISI	Inter-Stimuli Interval
P300 EP	P300 Evoked Potential
SBC	Single-board Computer
SSVEP	Steady-State Visual Evoked Potential
SVM	Support-Vector Machine
UI	User Interface
VOG	Videooculography
