# Supplementary material for: Proposals and Comparisons from One-Sensor EEG and EOG Human-Machine Interfaces
Source: Sensors (Basel). 2021 Mar 22;21(6):2220. doi: 10.3390/s21062220 (PMC8004835; doi:10.3390/s21062220)
Supplement: Supplementary file 1 [file sensors-21-02220-s001.zip › SupplementaryMaterial/ExampleCode.zip!/README.pdf]

Proposals and Comparisons from One-Sensor EEG and EOG Human-Machine Interfaces

Francisco Laport*, Daniel Iglesia, Adriana Dapena, Paula M. Castro and Francisco J. Vazquez-Araujo

CITIC Research Center, University of A Coruña, Campus de Elviña, 15071 A Coruña, Spain;

* Correspondence: francisco.laport@udc.es

Introduction

This document aims to describe the recorded data and the example code provided with the published paper. For this purpose, it is important to remember that 4 experiments were carried out. Two of them using the P300 evoked potential as the control signal and the remaining two using blinking movements as control signals. Moreover, for each control signal, two paradigms were compared: home elements are presented one-by-one; (ii) the elements of the same row/column are presented together.

For each experiment, 8 different recordings were made, each one stored in a different folder. Therefore, in total there are 32 folders (4 experiments x 8 recordings). The data provided is organized in separate folders, whose name indicates: the participant, the control signal, the paradigm used and the recording number. For example, the folder ***P1-P300-1by1-R1*** indicates that the data is from participant 1, obtained for the P300 control signal, with the 1 by 1 paradigm and its first recording.

In each folder you will find 3 CSV files containing information related to the recording:

- **ExpInfo.csv:** contains information of the recording such as the sampling frequency, the target element, inter-stimulus time, etc.
- **RAW-EEG.csv:** contains the raw data obtained by the Cyton board. There are 13 columns whose name can be seen in the first row of the file. The numbers 1 through 8 indicate the channels on the Cyton board. Columns A5, A6 and A7 indicate data obtained by the analogue pins of the board. The two important columns in this file will be the column with the information of channel 1 since it is the one that contains the EEG / EOG signal of the recording; and column A6 since it contains the information of the triggers, i.e., it tells us when a stimulus has been presented.
- **RAW-STIMS.csv:** it contains information related to the stimuli presented to the user. It is made up of 5 columns, whose names can be seen in the first row. The important column will be the Stims column, since it contains the codes of the stimuli presented to the user. All codes above 100 indicate a presented stimulus. Codes below 100 are control codes (start end recording, etc.). Therefore, we will only focus on codes greater than 100.

In this example you will only find the data corresponding to participant 1. If you wish to obtain the data of the rest of the participants, please contact Francisco Laport through the email provided above.

Stimuli codes

Each stimulus presented to the user is defined in the RAW-STIM.csv file by a specific code. This code will be different for each paradigm. In the case of 1 by 1, the stimulus codes will go from

101 to 109, one for each element of the matrix (see figure 1.a), in the case of the row-column, codes will go from 101 to 106, one for each row and column (see figure 1.b).

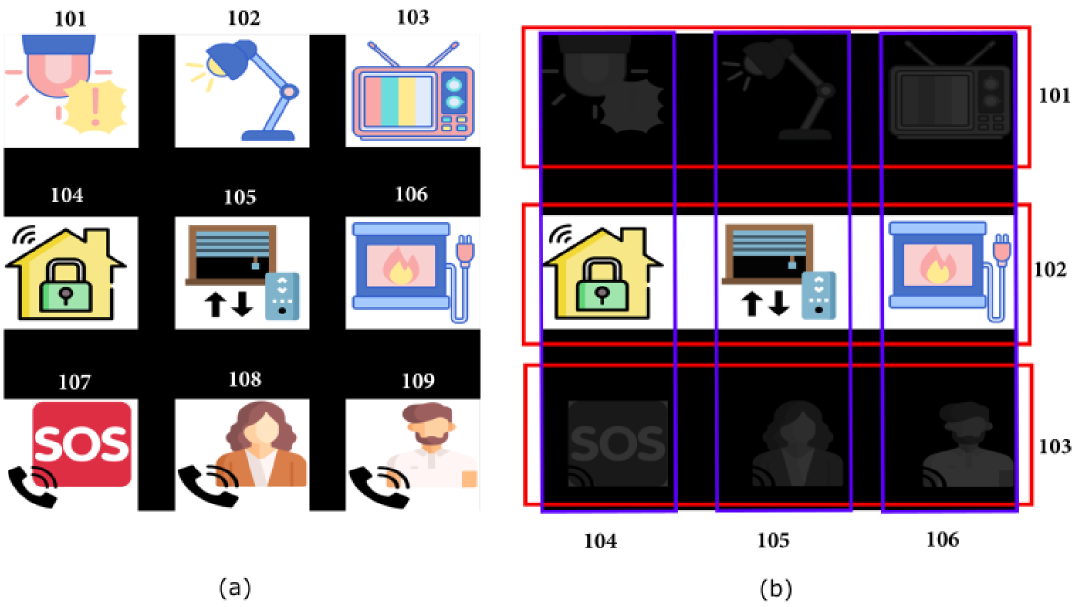

Figure 1. Stimuli codes for 1-by-1 paradigm (a) and Row-column paradigm (b).

Trigger detection

To achieve good synchronization between the data obtained by the Cyton board and the stimuli presented by the GUI, a photoresistor cell was used as an external trigger. The data from this cell is captured by the analog pin A6 of the Cyton board. This cell is connected to the screen of the experiments, therefore, each time a stimulus (with a code greater than 100) is presented to the user, the intensity of the area where the cell is connected increases. In this way, each peak that we find in the signal captured by the photoresist cell corresponds to the presentation of a stimulus (see figure 2).

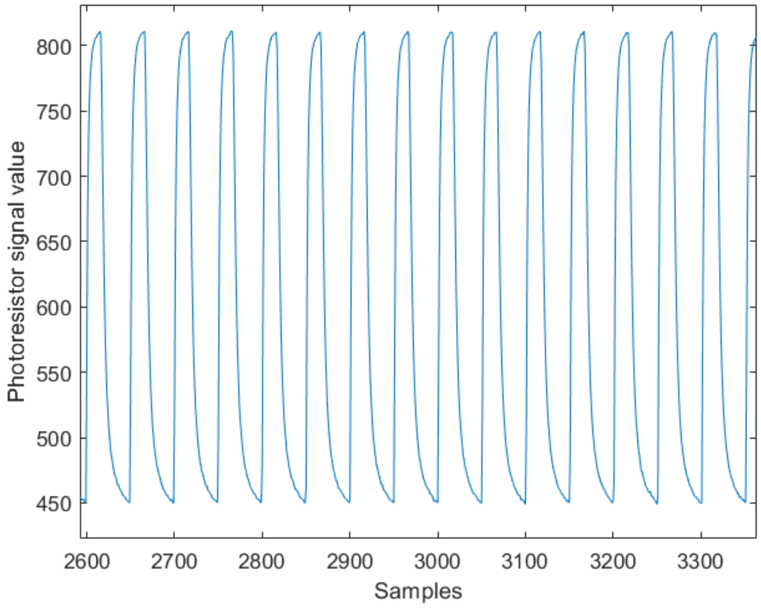

Figure 2. Signal captured by the photoresistor cell. Each peak corresponds to the presentation of one stimulus.
